# The influence of bisphosphonates on human osteoblast migration and integrin aVb3/tenascin C gene expression in vitro

**DOI:** 10.1186/1746-160X-7-4

**Published:** 2011-02-07

**Authors:** Felix P Koch, Annette Wunsch, Christina Merkel, Thomas Ziebart, Andreas Pabst, Sareh Said Yekta, Marco Blessmann, Ralf Smeets

**Affiliations:** 1 Department of Oral and Maxillofacial Surgery, University medical centre of the Johannes Gutenberg University Mainz, Augustusplatz 2, 55131 Mainz, Germany; 2 Department of Oral and Maxillofacial Surgery, University Hospital Aachen, Pauwelsstraße 30, 52074 Aachen, Germany; 3 Department of Oral and Maxillofacial Surgery, University Medical Center Hamburg-Eppendorf, Martinistraße 52, 20246 Hamburg, Germany

## Abstract

**Background:**

Bisphosphonates are therapeutics of bone diseases, such as Paget's disease, multiple myeloma or osteoclastic metastases. As a severe side effect the bisphosphonate induced osteonecrosis of the jaw (BONJ) often requires surgical treatment and is accompanied with a disturbed wound healing.

Therefore, the influence on adhesion and migration of human osteoblasts (hOB) after bisphosphonate therapy has been investigated by morphologic as well as gene expression methods.

**Methods:**

By a scratch wound experiment, which measures the reduction of defined cell layer gap, the morphology and migration ability of hOB was evaluated. A test group of hOB, which was stimulated by zoledronate 5 × 10^-5^M, and a control group of unstimulated hOB were applied. Furthermore the gene expression of integrin aVb3 and tenascin C was quantified by Real-Time rtPCR at 5data points over an experimental period of 14 days. The bisphosphonates zoledronate, ibandronate and clodronate have been compared with an unstimulated hOB control.

**Results:**

After initially identical migration and adhesion characteristics, zoledronate inhibited hOB migration after 50 h of stimulation. The integrinavb3 and tenascin C gene expression was effected by bisphosphonates in a cell line dependent manner with decreased, respectively inconsistent gene expression levels over time. The non-nitrogen containing bisphosphonates clodronate led to decreased gene expression levels.

**Conclusion:**

Bisphosphonates seem to inhibit hOB adhesion and migration. The integrin aVb3 and tenascin C gene expression seem to be dependent on the cell line. BONJ could be enhanced by an inhibition of osteoblast adhesion and migration. The gene expression results, however, suggest a cell line dependent effect of bisphosphonates, which could explain the interindividual differences of BONJ incidences.

## Background

Bisphosphonates are proven to be effective in the treatment of benign or malignant skeletal diseases characterized by enhanced bone resorption. They are comprised of two phosphate groups, which are capable of binding divalent ions, such as Mg^2+ ^and Ca^2+^. This activity is the basis of bone targeting of these therapeutics. These effects, however, also seem to be dependent on the PH value [1]. Bisphosphonates not only inhibit proliferation and induce apoptosis in cultured cancer cells, but additionally interfere with adhesion of cancer cells and osteoblasts to the bone matrix and inhibit cell migration and invasion [2,3]. The influence on cell migration and adhesion could also affect the wound healing potency of osteoprogenitor cells after dentoalveolar surgery and promote the side effect of BONJ.

Osteoblast migration is regulated by several surface proteins, such as integrins. These are responsible for cell adhesion as well as cell signaling [4,5]. Another recently discussed substrate is tenascin C, which seems to influence the function of other adhesion proteins such as adhesins and syndecans [6]. Tenascin as well influences cell shape and inhibits the activity of the focal adhesion kinase, an intracellular protein binding the integrins and accumulating vinculin, talin and α-actinin in the focal adhesion site [6-8]. The function of tenascin C for cell adhesion, however, is controversially discussed. There are reports of a stimulation as well as inhibition effect on cell adhesion and migration [9-11]. Tenascin C knockout mice showing an altered fracture healing, it seems to be an important regulator of osteoblast migration during wound healing [12].

Since adhesion and migration are important factors of wound healing, this study aimed to investigate the influence of zoledronate on osteoblast by morphologic cell culture experiments as well as integrin aVb3 and tenascin C gene expression.

## Materials and methods

### Cell migration tests

#### - Scratch Wound

Human osteoblasts (HOB-c, Promo Cell, Heidelberg, Germany) between passages 5-7 were cultured on 6-well plates, covering the whole bottom of the well after five days of cultivation. A defined scratch was applied on the well bottom, which detached cells within a definite corridor. Over a time period of five days the percental recovering of the scratch wound was monitored.

### Gene expression analysis

#### - Cell culture

Human osteoblasts (HOB-c, Promo Cell, Heidelberg, Germany) between passages 5-7 were cultured at a density of 200 000 cells per well using 6-well plates. They were allowed to attach for two days using an osteoblast specific medium (10% FCS/DMEM Dulbecco modified medium (Invitrogen, Carlsbad, Ca/US) containing 1% L-glutamin, 1% penicillin/streptomycin/neomycin, 1% ascorbic acid, and 20 μg/ml dexamethasone. After the attachment phase, the cells were stimulated by osteoblast specific medium containing zoledronate, ibandronate or clodronate at a concentration of 5 × 10^-5^M. The osteoblast specific cell culture medium without bisphosphonate supplement was used for control. The media and bisphosphonates were renewed every 4 days for a period of 14 days to guarantee a constant stimulation und nutrition supply over the experimental period.

#### - mRNA extraction and reverse transcriptase polymerase chain reaction (RT-PCR)

On day 1, 2, 5, 10, and 14 of cultivation, the osteoblasts were detached with 0.05% trypsin-EDTA solution (Invitrogen, Carlsbad, Ca, US) and individually harvested. mRNA was extracted using a silicate gel technique that was provided by the Qiagen RNeasy extraction kit (Qiagen, Hilden, Germany). This included a DNAse digestion step. The amount of extracted mRNA was measured by extinction at 260 nm; the contamination with proteins was determinated with the 260/280 ratio.

To detect the mRNA of integrin aVb3 and tenascin C in osteoblasts, primers were designed using NCBI-nucleotide library and Primer3-design (Table 1). All primers had been matched to the mRNA sequences of the target genes (NCBI Blast software).

**Table 1:** Oligonucleotide primer sequences used for Real Time PCR

	Sense	Antisense
**Integrin aVb3**	TTGTTTCAGGAGTTCCAAGA	TGAAGAGAGGTGCTCCAATA

**Tenascin C**	GAGACATCTGTGGAAGTGGA	CGTACTCAGTGTCAGGCTTC

**GAPDH**	AAAAACCTGCCAAATATGAT	CAGTGAGGGTCTCTCTCTTC

**RPS 18**	TCGGAACTGAGGCCATGA	GAACCTCCGACTTTCGTTC

**Actin**	GGAGCAATGATCTTGATCTT	CCTTCCTGGGCATGGAGTCCT

As housekeeping genes, human ribosomal protein (HuPO), actin, glyceraldehyde-3-phosphate dehydrogenase (GAPDH) and ribosomal protein S18 (RPS18) were evaluated. We were able to show the most stable expression for the actin, GAPDH and RPS18 genes by comparing the bisphosphonate stimulated versus a non stimulated cell-culture using a specialized freeware, called GeNorm.

As a quantitative RT-PCR we used the SYBR Green Real Time PCR (oneStep RT-PCR, Bio-Rad, Hercules, CA/USA). This method enables reverse transcription using the individual primers immediately before PCR amplification and SYBR Green fluorescence measurement for quantification of gene expression. Samples were amplified in 96-well microplates in an IQ5-Cycler (Bio-Rad, Hercules, CA/USA) with an annealing temperature of 56°C and an elongation temperature of 71°C over 40 cycles. Background was to determine over 3-10 cycles and the threshold were set above this fluorescence, crossing the SYBR green fluorescence curve at the exponential part. This method was applied to calculate the cycle number and C_T_-value for quantitation. Furthermore, the C_T_-values of actin, GAPDH and RPS18 housekeeping genes and the individual primer efficacy were considered. Single product formation was confirmed by melting point analysis. For negative control, water instead of mRNA-samples was used.

CDNA from individual cell experiments was analyzed in triplicate PCR. The ΔΔC_T _method was applied [13,14] for a statistical analysis of the C_T_-values. For each specific primer and Real-Time PCR, the efficiency was calculated on the basis of the SYBR Green fluorescence curves and the standard dilution series. The relative gene expression levels were standardized with those measured in the unstimulated control, which was set to 100%. Each point in time for relative mRNA is the mean +/- standard deviation.

### - Statistical analysis

The mean values and standard deviations were calculated by the IQ5-software (BioRad, Hercules, CA/USA) to provide a descriptive data analysis.

## Results

### Scratch wound

**Figure 1:**
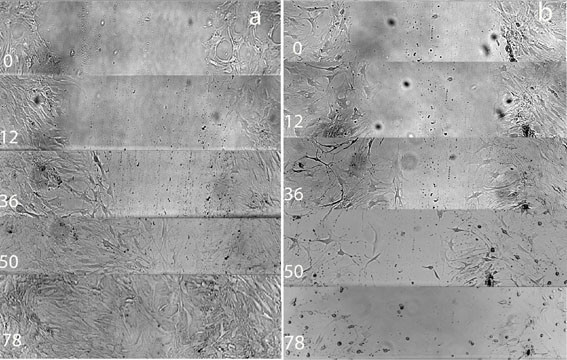
**Synopsis of scratch wound microscopic images during the first 78 h: (a) control, (b) stimulation by zoledronate 5 × 10^-5^M**. (Magnification 10×).

Figure 1 shows microscopic pictures of the experiments 12, 36, 50 and 78 hours after the scratch wound setting. The morphologic changes of the attached cells as well as the differing width of the scratch wounds are presented. Compared to the unstimulated control, zoledronate changed the typical dendritic osteoblast morphology to spherical appearance. The cellular gap was opened after an intermediate closure after 50 h again (Figure 1).

**Figure 2:**
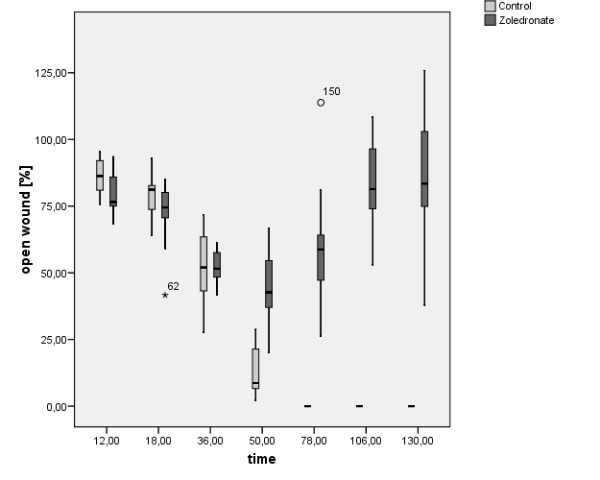
**Box plot graph of the scratch wound gap over the experimental period of 130 h**. (relative scratch wound gap compared to the initial gap).

The gap of the scratch wound compared to the width of the gap at the beginning of the experiment is demonstrated by Figure 2. Both, the hOBs of the control as well as the zoledronate group, tended to close the scratch wound gap during the first 36 h. After 50 h, however, the gap was not further reduced in the zoledronate group, while the scratch wound gap has been completely recovered after 78 h in the control group (Figure 2a &2b).

### Integrin aVb3 gene expression

**Figure 3:**
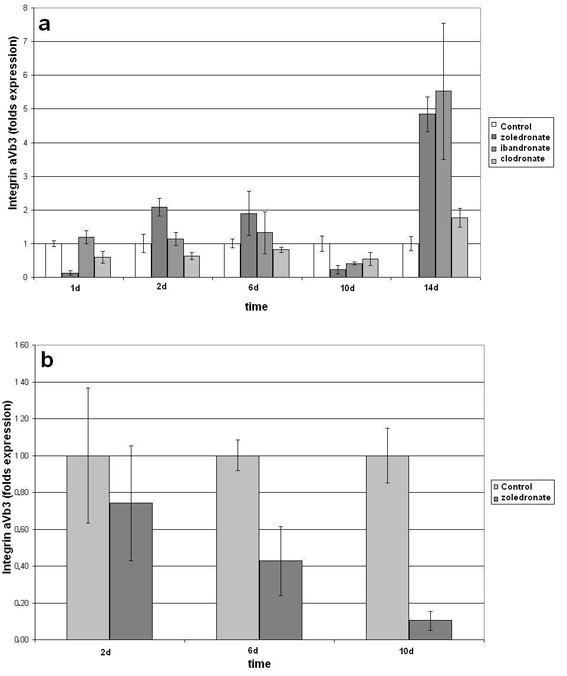
**Quantitative RT-PCR-results of integrin aVb3 gene expression as fold of unstimulated control gene expression (means +/- SD), that was set at 1 (100%)**. (a & b are due to different hOB cell lines).

With respect to the different hOB cell lines, integrin aVb3 was expressed at different levels. Furthermore, the integrin aVb3 gene expression was influenced in a time dependent manner. Figure 3a shows an enhanced gene expression at day 2, 6 and 14 with a maximum of 500% after stimulation by zoledronate and ibandronate, whereas at day 1 and 10 the gene expression was decreased compared to the unstimulated control. The integrin gene expression of another cell line is presented in Figure 3b. At all data points the gene expression was lower compared to control after stimulation with zoledronate.

The non-nitrogen containing clodronate was expressed at lower levels during the first 10 days and caused as well a higher gene expression at day 14 compared to control (Figure 2a; Table 2a).

**Table 2:** Detailed integrin aVb3 (a) and tenascin C (b) gene expression results of cell culture 1 (C1) and cell culture 2 (C2).

a. integrin aVb3	1d	2d	6d	10d	14d
**Zoledronate (C1)**	-	0,74 (±0,31)	0,43 (±0,19)	0,10 (±0,05)	-

**Zoledronate (C2)**	0,13 (±0,07)	2,08 (±0,26)	1,89 (±0,66)	0,23 (±0,12)	4,84 (±0,51)

**Ibandronate (C2)**	1,19 (±0,19)	1,14 (±0,19)	1,32 (±0,62)	0,41(±0,04)	5,52 (±2,02)

**Clodronate (C2)**	0,60 (±0,17)	0,64 (±0,11)	0,81 (±0,09)	0,54 (±0,19)	1,77 (±0,27)

**b. tenascin C**	**1d**	**2d**	**6d**	**10d**	**14d**

**Zoledronate (C1)**	-	0,31 (±0,1)	0,06 (±0,01)	0,04 (±0,004)	-

**Zoledronate (C2)**	4,93 (±0,81)	0,59 (±0,04)	n.a.	0,07 (±0,04)	9,37 (±0,83)

**Ibandronate (C2)**	0,14 (±0,03)	0,46 (±0,08)	n.a.	0,86 (±0,08)	13,12(±2,9)

**Clodronate (C2)**	0,93 (±0,34)	0,38 (±0,06)	n.a.	0,26 (±0,09)	1,43 (±0,37)

### Tenascin C gene expression

**Figure 4:**
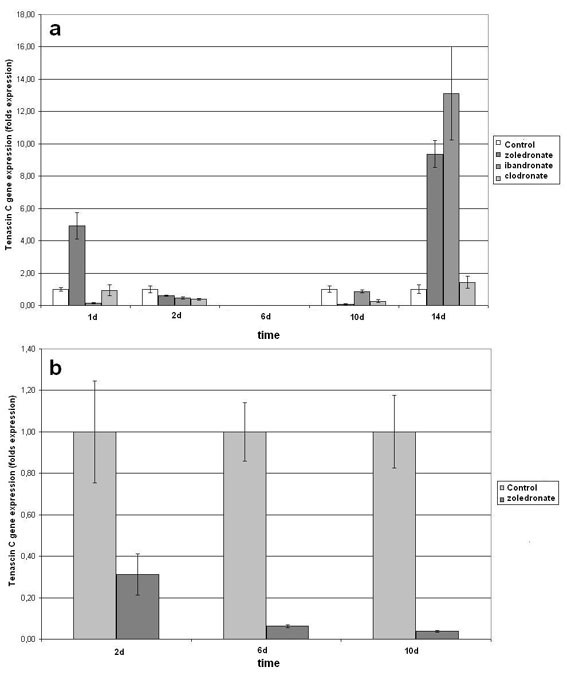
**Quantitative RT-PCR-results of integrin aVb3 gene expression as fold of unstimulated control gene expression (means +/- SD), that was set at 1 (100%)**. (a & b are due to different hOB cell lines).

Except for the first day with zoledronate stimulation and day 14, tenascin C was expressed at lower levels after zoledronate and ibandronate stimulation. Both cell lines were affected by bisphosphonates in the same way (Figure 4a, Figure 4b). At day 14, tenascin C gene expression was evidently enhanced.

The non-nitrogen containing bisphosphonate clodronate caused as well a decreased gene expression during the first 10 experimental days. Tenascin C, however, was not significantly enhanced at day 14 compared to the unstimulated control (Figure 4a; Table 2b).

## Discussion

The migration of osteogenic cells is a crucial step during physiological wound healing. This regulation seems to be disturbed after application of bisphosphonates, as most BONJ occur after surgery of the oral cavity.

The data of our study suggest an inhibition of hOB migration and disturbance of cell morphology by bisphosphonates.

With respect to other studies, this is in agreement with the inhibition of metastases ingrowths and tumor cell migration. Additionally to published studies on hOB migration, which could as well show the inhibition of migration, by morphologic methods, this work gives insight in the cellular mechanisms responsible for adhesion and migration. For the first time the effect of bisphosphonates on integrin aVb3 and tenascin C gene expression has been quantified in hOBs.

Integrin aVb3 plays an important role in focal adhesion, intercellular communication and differentiation. Overall, it regulates cell migration and adhesion, stimulated by extracellular ligands as laminin, fibronectin and tenascin.

Tenascin C regulates OB focal adhesion by interacting with extracellular proteins and cellular receptors as integrins [15-17]. It is elevated during wound healing and tumor invasion [18]. As differential local detachment and reattachment of cells to the extracellular matrix is required during cell growth and wound healing, tenascin C seems to have adhesive and antiadhesive functions [19,20], possibly depending on isoforms produced by alternative splicing [21].

Corresponding to literature and our morphologic findings, the downregulation of the adhesive genes integrin aVb3 and tenascin C, which possibly even enhanced the antiadhesive effect by autocrine secretion, could be one of the molecular, intracellular reasons for the BONJ. Obviously this effect depends on the cell line, which showed decreased as well as increased integrin and tenascin gene expression levels. This effect of bisphosphonates could possibly explain the interindividual differing BONJ incidence. Even if risk factors as radiation or chemotherapy are not present, BONJ could occur as individual predisposition. The enhancement of integrin aVb3 gene expression after 14 days could be due to the induction of apoptotic cell death. There is evidence, that cell fate is regulated at least in part through membrane proteins, such as integrins, which send signals to the nucleolus and regulate gene expression leading to apoptosis.

## Competing interests

The authors declare that they have no competing interests.

## Authors' contributions

FK conceived of the study, organized and carried out the parts of the PCR studies, designed the primers and drafted the manuscript. AW carried out the scratch wound experiments. CK carried out the PCR studies. TZ and AP contributed their experience of scratch wound experiments. SSY, MB and RS participated in the study design, supported by scientific consulting and coordination and helped to draft the manuscript. All authors read and approved the final manuscript.
